# Comparing open and robot-assisted partial nephrectomy – a single institution report

**DOI:** 10.1186/s12894-024-01586-6

**Published:** 2024-09-09

**Authors:** Marius Roaldsen, Vetle Lohne, Thor Allan Stenberg, Hiten R.H. Patel, Erling Aarsaether

**Affiliations:** 1 University Hospital of North Norway, Tromsø, Norway; 2 UiT – the arctic University of Norway, Tromsø, Norway

**Keywords:** Nephrometry score, Nephron sparing surgery, Partial nephrectomy, Renal cell carcinoma, Renal ischemia reperfusion injury, Robot-assisted surgery

## Abstract

**Background:**

Open partial nephrectomy (OPN) has previously been considered the gold standard procedure for treatment of T1 localized renal tumors. After introduction of robot assisted partial nephrectomy (RAPN) as an alternative method to OPN, OPN was gradually abandoned at our department. The aim of the study was to retrospectively compare the results of patients treated with either OPN or RAPN for suspected renal carcinoma.

**Methods:**

Patients who underwent either open or robotic assisted partial nephrectomy between January 1st 2010 and December 31st 2020 were retrospectively included in the study. Each tumor subjected to surgery was scored preoperatively by the RENAL nephrometry score. Complications within 30 days were assessed according to the Clavien-Dindo classification system.

**Results:**

A total of 197 patients who underwent partial nephrectomy were identified; 75 were subjected to OPN and 122 were treated with RAPN. There were no significant differences between the groups with respect to age (OPN: 63 years ± 11, RAPN: 62 years ± 10), gender (OPN: 71/29%, RAPN: 67/33%), body mass index (OPN: 28 ± 5, RAPN: 28 ± 5), ASA score (OPN: 2.4 ± 0.6, RAPN: 2.2 ± 0.5), or nephrometry score (OPN: 6.6 ± 1.7, RAPN: 6.9 ± 1.7, *p* = 0.2). The operative time was significantly shorter in the OPN group (81 min) compared to the RAPN group (144.5 min, *p* < 0.001). Mean perioperative blood loss was 227 ± 162 ml in the OPN group compared to 189 ± 152 ml in the RAPN group (*p* = 0.1). Mean length of stay was shorter in the RAPN group (3 days) compared to the OPN group (6, days, *p* < 0.001). Positive surgical margin rate was significantly higher in the OPN group (21.6%) compared to the RAPN group (4.2%, *p* < 0.001). There were no differences in the number of Clavien-Dindo graded complications between the groups (*p* = 0.6).

**Conclusions:**

The introduction of RAPN at our department resulted in shorter length of stay and fewer positive surgical margins, without increasing complications.

## Introduction

Open partial nephrectomy (OPN) has previously been considered the gold standard for treatment for T1 localized renal tumors [1, 2], but the procedure is associated with perioperative morbidity and longer hospital stays when compared to minimally invasive procedures [3]. Laparoscopic partial nephrectomy (LPN) was first published as a case report in 1993, in which the authors emphasized the benefit of improved postoperative pain management and recovery as a clear advantage of this technique [4]. Today, LPN as a procedure demonstrates comparable oncologic results to that of OPN [5]. Robotic assisted partial nephrectomy (RAPN), which was introduced in 2004, offers similar minimally invasive advantages to that of LPN, but increases operative visualization by three-dimensional imaging, the ability to control the camera without an assistant and improved dexterity through flexible instruments that mimics the function of the human hand [6]. In addition to superior visual and instrument control, RAPN has been shown to demonstrate shorter learning curve compared to LPN [7, 8]. After the successful introduction of robotic assisted partial nephrectomy (RAPN) at our department in 2014, the open partial nephrectomy procedure was gradually abandoned. RAPN was established as the standard procedure, although without evidence of RAPN as a method being superior to or even equal in quality to that of OPN. The aim of the study was to retrospectively compare the outcomes of patients who underwent partial nephrectomy by either open or robotic assisted partial nephrectomy at our department.

## Materials and methods

The study was approved by the Regional Ethical Committee at the University Hospital of North Norway. Inclusion critera were the following: Patients over 18 years of age who underwent either open or robotic assisted partial nephrectomy of a suspected renal cell carcinoma between January 1st 2010 and December 31st 2020. The patients were identified by a search for the procedure codes of either OPN or RAPN in the electronic journal system (DIPS, Distribuert Informasjons- og Pasientdatasystem i Sykehus, Bodoe, Norway). Exclusion criteria were the following: Patients who had simultaneous surgery of other organs in the same surgical procedure, or patients with erroneous procedure codes; i.e. mismatch between procedure code and the actual surgical procedure. Patients who were converted from RAPN to OPN were also excluded from the study. All patients were retrospectively included in the study.

In the OPN group access to the kidney was achieved through a flank or anterior subcostal incision at the individual surgeon`s preference. The Gerota`s fascia was opened; the tumor was localized, and the renal vessels were secured with vessel-loops [2]. Intraoperative ultrasonography was not routinely utilized before tumor resection. The tumor was resected bluntly, and the renal artery was not clamped, unless bleeding control was considered inadequate.

In the RAPN group the procedures were performed with a standardized 4-arm transperitoneal 6-port approach using a 30° lens, a fenestrated bipolar forceps, monopolar curved scissors and a ProGrasp™ forceps (Intuitive Surgical Inc., Sunnyvale, CA, USA). The da Vinci SI^®^ (Intuitive Surgical Inc., Sunnyvale, CA, USA) system was utilized from 2014 to 2019, while the subsequent cases were performed with the da Vinci XI^®^ (Intuitive Surgical Inc., Sunnyvale, CA, USA). After removal of perinephric fat, the tumor was visualized by intraoperative ultrasound in all procedures. In contrast to the procedures in the OPN group, the renal artery was clamped in all cases.

Clinical data were manually extracted from the available patient records (Table 1). Preoperative computed tomography scans of each tumor subjected to surgery were scored retrospectively by the RENAL nephrometry score [9]. Glomerular filtration rate (GFR) was calculated from serum creatinine and corrected for age and gender. The GFR was measured preoperatively and 7:30 AM every postoperative day until patients were discharged. Postoperative GFR was defined as the single lowest observed value in the postoperative period. The 30-day complication rate was retrospectively collected and graded according to the Clavien-Dindo classification system [10]. Statistical analysis was performed with the IBM SPSS software (Chicago, Ill). Comparisons of numerical variables between the groups were calculated with the t test. Between-group comparisons for categorical variables were analyzed with the Pearson chi square test or the Exact test, when one or more cells in the computed 2 × 2 table displayed expected cell counts of less than five. *P* < 0.05 was considered statistically significant.

**Table 1:** Preoperative data

	OPN (*n* = 75)	RAPN (*n* = 122)	*p*
Age (years)	63 ± 11	62 ± 10	0.6
Gender, % (male/female)	71/29	67/33	0.5
BMI	28.4 ± 5	27.8 ± 5	0.4
ASA score	2.4 ± 0.6	2.2 ± 0.5	0.07
RENAL nephrometry score	6.6 ± 1.7	6.9 ± 1.7	0.2

ASA = American Society of Anesthesiologists, BMI = Body mass index, OPN = Open partial nephrectomy, RAPN = Robot assisted partial nephrectomy

## Results

A total of 197 patients who underwent partial nephrectomy were identified. Preoperative data from the patients are summarized in Table 1. A number of 75 patients underwent OPN, while 122 patients were treated with RAPN. The two patient’s groups were similar with respect to age, gender, body mass index, and ASA score. Nephrometry score was 6,6 ± 1,7 in the OPN group compared to 6,9 ± 1,7 in the RAPN group (*p* = 0,2). A summary of the collected peri- and postoperative data is presented in Table 2. There was no difference in preoperative GFR between the groups. Mean ischemia time was shorter in the OPN group compared to the RAPN group, but the difference was not statistically significant. However, 68 of the 75 surgical procedures in the OPN group were performed off-clamp. The ischemia time in this group therefore only reflects the mean time from 7 cases, in which the renal artery was actually clamped. In comparison, the renal artery was clamped in all 122 cases in the RAPN group. There was no significant difference in either postoperative GFR or a mean change in GFR from baseline between the groups. The operative time was significantly shorter in the OPN group (81 min) compared to the RAPN group (144.5 min, *p* < 0.001). Mean perioperative blood loss was 227 ± 162 ml in the OPN group compared to 189 ± 152 ml in the RAPN group (*p* = 0.1), while the mean length of stay was 3 days in the RAPN group compared to 6 days in the OPN group (*p* < 0.001). Positive surgical margin rate was significantly higher in the OPN group (21.6%) compared to the RAPN group (4.2%, *p* < 0.001).

**Table 2:** Peri- and postoperative data

	OPN (*n* = 75)	RAPN (*n* = 122)	*p*
Preoperative GFR (ml/min/1.73m^2^)	84 ± 19	85 ± 17	0.7
Ischemia time (min.)	10.4 ± 4*	14.6 ± 6	0.08
Postoperative GFR (ml/min/1.73m2)	72 ± 22	70 ± 20	0.7
GFR mean difference (ml/min/1.73m2)	12 ± 11	14 ± 14	0.3
Operative time (minutes)	87 ± 27	152 ± 47	< 0.001
Blood loss (ml)	227 ± 162	189 ± 152	0.1
Postoperative length of stay (days)	6.0 ± 2.5	3.6 ± 2.6	< 0.001
Positive surgical margins (%)	21.6	4.2	< 0.001

* Ischemia time was only provided from seven procedures (*n* = 7) in the OPN group, while the rest of the procedures (*n* = 68) were performed off-clamp. GFR = Glomerular filtration rate

Complications within 30 days are displayed in Table 3. According to the Clavien-Dindo classification system, complications of any grade were present in 33.3% of patients in the OPN group compared to 30.1% in the RAPN group (*p* = 0.6). Grade II complications, such as treatment with antibiotics or blood products were most frequently observed. In the OPN group a grade II complication was documented in 14.7% of the patients compared to 13.8% in the RAPN group (*p* = 0.9). The more serious complications, grade III and IV, were much less frequently observed. There were no significant differences with respect to complications between the two groups.

**Table 3:** Complications within 30 days

Clavien-Dindo *n* (%)	OPN (*n* = 75)	RAPN (*n* = 122)	*p*
Grade I	9 (12%)	15 (12.2%)	1.0
Grade II	11 (14.7%)	17 (13.8%)	0.9
Grade IIIa	1 (1.3%)	3 (2.4%)	1.0
Grade IIIb	3 (4%)	2 (1.6%)	0.4
Grade IVa	1 (1.3%)	nil	0.4
Grade IVb	nil	nil	-
Grade V	nil	nil	-
All	25 (33.3%)	37 (30.1%)	0.6

OPN = Open partial nephrectomy, RAPN = Robot assisted partial nephrectomy

## Discussion

Our study results are in agreement with similar studies, which have compared OPN to RAPN, indicating longer operative time for RAPN, but shorter length of stay [11]. While previous studies have shown less estimated bleeding in patients undergoing RAPN compared to OPN, the difference in bleeding between the groups did not reach statistical significance in our study. However, less bleeding has been repeatedly documented for patient series comparing OPN and RAPN [11, 12]. It has been hypothesized that the relatively lower bleeding volumes demonstrated by RAPN is due to the superior vision and instrument control provided by the da Vinci surgical system, which limits bleeding and allows for selective coagulation [3, 6]. We underline that the learning curve of RAPN is included in the data provided in the present study. While OPN was well established as the state-of-the-art surgery in the period of time from which these data were collected, the data in the RAPN group contains the very first procedures performed at our department. Although the steepness of the learning curve of RAPN probably relies on individual talent, skills and previous experience, a minimal number of 30 procedures has been suggested by Mottrie and others in order to master RAPN as a method [13]. However, in a later publication by the same group, the relationship between experience and warm ischemia time displayed a steep slope reduction within the first 100 cases and did first reach a plateau after 150 cases, while the learning curve for complications failed to reach a plateau, even after 300 cases [14]. We therefore speculate that estimated bleeding would have been significantly lower in the RAPN group, if more patients had been included the study.

Warm ischemia time is often considered a measure of the renal injury sustained during partial nephrectomy, regardless of whether the surgery is performed as an open or minimally invasive procedure. However, one important difference between the study groups is that the majority of the procedures in the OPN group was achieved without clamping the renal artery. In comparison, the renal artery was clamped in all the cases in the RAPN group, in order to complete the resection of the tumor. During an open off-clamp partial nephrectomy the bleeding may be controlled by gently applying manual pressure on the edges of the resected renal parenchyma. Although off-clamp partial nephrectomy may also be performed robotically, the lack of tactile feedback from the daVinci system makes it more difficult to control bleeding by applying direct pressure to the kidney in this setting. However, other techniques such as the application of multiple ultrasound-guided stitches in the tumor bed before resection has been described with excellent results [15].

The postoperative drop in GFR commonly observed within the first 48 h after partial nephrectomy is frequently utilized as evidence of acute kidney injury. A postoperative decline in GFR was also observed in both groups in the present study, but there was no significant difference between the groups. Excluding the seven on-clamp cases in the OPN group decreased the mean drop in GFR from 12 ± 11 to 11 ± 11 ml/min/1.73m^2^, indicating a small but significant correlation between the postoperative drop in GFR and warm ischemia time (Pearson correlation 0.29, *p* < 0.05). In general, assessment of acute kidney injury in partial nephrectomy represents a challenge due to the lack of an adequate biomarker [16]. A biomarker of tubular injury is warranted since the kidney tubule is the most metabolically active segment of the nephron and therefore uniquely susceptible to ischemic and nephrotoxic insults [16, 17]. We previously utilized histological injury score, urinary albumin and urinary albumin/creatinine ratio in order to distinguish the acute kidney injury between two groups of pigs which both exhibited a sixfold rise in serum creatinine following renal ischemia reperfusion injury [18].

An interesting finding in the present study is the significant lower positive surgical margin rate observed in the RAPN group compared to the OPN group. Although several studies have indicated fewer positive surgical margins in RAPN when compared to OPN [11, 12, 19–22], the present study represents only the second report to our knowledge of statistically significant differences in favor of RAPN compared to OPN with respect to positive surgical margin rate. Tan and others reported positive surgical margins of 10.9% in the OPN group and 3.5% in the RAPN group respectively, in a study of similar size, which also included the learning curve of the cases in the RAPN group [23]. Positive surgical margins usually occur in 2–8% of partial nephrectomies and may reflect the quality of the surgery. The reason for the unexpected high positive surgical margin rate in the OPN group in the present study is not known. Both low surgeon volume, which has been suggested as a risk of positive surgical margins by some authors [24], or the relatively small sample size are possible explanations. In general, great effort should be invested in avoiding positive surgical margins in partial nephrectomy, since it at least in theory raises the question of whether cancerous tissue remains in the resection bed [25]. A recently published meta-analysis based on 39 studies found no difference in survival among partial nephrectomy patients with positive surgical margins, although an increased risk of recurrence and metastatic disease was reported [26].

Study limitations include the small sample size, retrospective design, lack of randomization and the unequal number of patients in each group. The fact that the learning curve is included in the RAPN group supports the case for RAPN as an excellent concept for nephron sparing surgery, also in a department of relatively low volume.

## Data Availability

The data that support the findings of this study are not openly available due to reasons of sensitivity and are available from the corresponding author upon reasonable request. Data are located in controlled access data storage at the University Hospital of North Norway.

## Abbreviations

ASA: American Society of Anesthesiologists
GFR: Glomerular filtration rate
LPN: Laparoscopic partial nephrectomy
OPN: Open partial nephrectomy
RAPN: Robot assisted partial nephrectomy
